# Increased Pain Intensity Is Associated with Greater Verbal Communication Difficulty and Increased Production of Speech and Co-Speech Gestures

**DOI:** 10.1371/journal.pone.0110779

**Published:** 2014-10-24

**Authors:** Samantha Rowbotham, April J. Wardy, Donna M. Lloyd, Alison Wearden, Judith Holler

**Affiliations:** 1 School of Psychological Sciences, University of Manchester, Manchester, United Kingdom; 2 Institute of Psychological Sciences, University of Leeds, Leeds, United Kingdom; 3 Max Planck Institute for Psycholinguistics, Nijmegen, Netherlands; College of Medicine, Taiwan

## Abstract

Effective pain communication is essential if adequate treatment and support are to be provided. Pain communication is often multimodal, with sufferers utilising speech, nonverbal behaviours (such as facial expressions), and co-speech gestures (bodily movements, primarily of the hands and arms that accompany speech and can convey semantic information) to communicate their experience. Research suggests that the production of nonverbal pain behaviours is positively associated with pain intensity, but it is not known whether this is also the case for speech and co-speech gestures. The present study explored whether increased pain intensity is associated with greater speech and gesture production during face-to-face communication about acute, experimental pain. Participants (N = 26) were exposed to experimentally elicited pressure pain to the fingernail bed at high and low intensities and took part in video-recorded semi-structured interviews. Despite rating more intense pain as more difficult to communicate (*t*(25) = 2.21, *p* = .037), participants produced significantly longer verbal pain descriptions and more co-speech gestures in the high intensity pain condition (Words: *t*(25) = 3.57, *p* = .001; Gestures: *t*(25) = 3.66, *p* = .001). This suggests that spoken and gestural communication about pain is enhanced when pain is more intense. Thus, in addition to conveying detailed semantic information about pain, speech and co-speech gestures may provide a cue to pain intensity, with implications for the treatment and support received by pain sufferers. Future work should consider whether these findings are applicable within the context of clinical interactions about pain.

## Introduction

Pain is an experience familiar to most people, with survey studies estimating the prevalence of pain within the general population to be between 30% and 72% [1]–[3]. While pain is usually self-limiting and does not require treatment, there are some instances (e.g. when pain is severe or persistent) in which help and support may be sought from healthcare professionals or close friends and family, and pain is a frequent feature of medical consultations [4], [5]. Due to the private and subjective nature of pain, it is necessary for sufferers to *communicate* their experience if others are to be aware of its presence and nature. Health care professionals are encouraged to elicit information about various dimensions of pain, including intensity, location, type and sensation, onset and duration, previous treatments, associated symptoms and impact on activities [6]–[8]. That communication about these (and other) dimensions of pain is effective is particularly important within healthcare settings to enable appropriate clinical responses such as diagnosis, treatment and support to occur.

Pain communication can be conceptualised as a three stage process in which the internal pain experience (A) is encoded into a pain message (B), from which the recipient can draw inferences (C) about the pain [9]–[13]. In producing a pain message (B), speakers often utilise numerous modalities to communicate their experience, including speech, nonverbal pain behaviours, and co-speech gestures. Through speech it is possible to provide detailed descriptions of pain, including sensation, intensity and duration of pain, where the pain is located, and the emotional and functional impact of pain. Indeed, it is because of this ability to convey a wide range of information that speech is considered to be the ‘gold-standard’ for pain communication [8], [9], [14].

Nonverbal pain behaviours such as grimacing, position shifts, restricted movement, rubbing the painful area, and sighing are also produced during pain descriptions [12], [15]–[17], and can serve to signal the presence and intensity of pain. While these pain behaviours are produced with varying degrees of automaticity and intentionality [12] and can be categorised according to whether they serve a protective function (e.g. quickly withdrawing and rubbing the finger after trapping it in door) or a communicative function (e.g. saying ‘ow’ or grimacing) [16], [17], they provide a visible manifestation (B) of the pain experience (A) from which the observer can draw inferences (C) and are thus considered a form of ‘pain communication’.

Finally, recent research has revealed that co-speech gestures are also produced during pain communication [18]–[20]. Co-speech gestures are defined as the communicative movements of the hands, arms and other body parts that are spontaneously produced alongside speech [18]–[22]. These movements can be broadly categorised into representational and non-representational gestures [23], with the former conveying semantic information related to the content of speech (e.g. producing a circular gesture while saying “it was a large table”), and the latter serving pragmatic functions such as managing turn-taking, marking the delivery of information or structuring the discourse [24]–[27]. In the domain of pain communication, the focus has been on *representational* gestures as these have the capacity to convey information about the pain experience. These representational co-speech gestures differ from nonverbal pain behaviours (such as facial expressions and posture shifts) and other movements (such as playing with the hair or fiddling with a pen) in that they are tightly connected to the speech system, and frequently contribute *propositional* information relating to the content of speech [27], [28]. Within the context of pain communication, these co-speech gestures often convey detailed visual information about various aspects of the pain message, including its location, size and sensation, and frequently contribute additional information about pain that is not contained in the accompanying speech [18]–[20]. For example, a speaker may say “it just comes on really suddenly”, while producing a gesture in which the hands are rapidly clenched and unclenched, indicating a cramping sensation that is not alluded to in speech.

Taken together, the above suggests that in order to make inferences (C) about pain, healthcare professionals should attend to the information conveyed across the different modalities (speech, nonverbal pain behaviours and co-speech gestures). However, pain communication is a complex process and the multimodal pain message (B) may be influenced by a variety of factors, including the nature of the pain experience, the individual experiencing the pain, and the social and cultural context [9], [12], [13]. For example, a number of studies have demonstrated increased levels of verbal and nonverbal pain communication amongst those who catastrophize about pain [16], [29]–[32], while others have shown that depressed individuals engage in more facial displays of pain than those without depression [33], [34]. Pain communication can also be supressed or exaggerated depending on factors such as cultural norms, interests of the sufferer or reinforcement and conditioning [9], [12], [35], [36]. For example, Larochette and colleagues [36] found that children reported having both pretended to have pain and hidden their pain, with the most common reasons for pretending to have pain being to get attention, miss school, as a joke or to get their sibling in trouble, while reasons for hiding pain were to avoid embarrassment, to avoid worrying their parents or to be allowed to keep playing. Concerning social factors, studies indicate that people who are alone display more facial expressions of pain than those who are with a stranger [37], [38] but are more expressive in the presence of solicitous others [39] or those who may underestimate their pain [40].

Characteristics of the pain experience may also influence the communication of pain and the association between pain intensity and communication is one that has received particular attention. For example, a meta-analysis of 29 studies found a positive association between self-reported pain intensity and observed nonverbal pain behaviour [15], and chronic orofacial pain patients have been found to select more words on a pain questionnaire when pain is intense [41]. This is an interesting finding as it suggests that increased displays of nonverbal pain behaviours may serve as an additional cue to pain intensity that can be used alongside self-reported judgements of pain intensity provided verbally or by means of pain assessment tools. However, research has not considered the influence of pain intensity on spontaneous verbal communication or the production of co-speech gestures. Given that speech and co-speech gestures convey detailed information about pain, their production may increase when pain is intense due to a desire to communicate the experience as effectively as possible in order to receive help from healthcare providers or concerned others.

This paper reports a first attempt to consider the impact of pain intensity on communication, with a focus on both the production of speech and co-speech gestures. We hypothesised that people would enhance their communication (i.e., produce more speech and co-speech gestures) following a high-intensity than a low-intensity pain procedure. Due to the myriad of factors that impact upon pain communication, this study will use an acute, experimentally-induced pain experience (pressure pain to the fingernail bed), allowing for the manipulation of pain intensity, while keeping constant other factors, such as type, cause and duration of pain. The use of experimentally elicited pain also allows for a repeated-measures design, with all participants experiencing both high and low intensity pain, reducing inter-individual differences (such as catastrophizing, previous experiences of pain and pain communication) that might impact on pain communication across the two conditions. Experimentally elicited pain is well accepted within research settings and has been used in a number of studies considering factors that influence pain communication [31], [42], [43]. To further ensure that any differences between the high and low intensity conditions can be attributed to the intensity of the pain, we also asked participants to judge how difficult they found verbally communicating about the high and low intensity pain. It is reasonable to suspect that participants may produce less speech (and accompanying co-speech gestures, due to the tight integration of the two modalities) if verbal communication is difficult. Considering communication difficulty will therefore ease the interpretation of our findings.

## Materials and Methods

### Participants

Twenty-six undergraduate psychology students (21 female; 24 right-handed, Mean age  = 19 years) took part in return for course credit. All were native English speakers and none had suffered any known language impairment or taken part in a similar study.

### Design

A within-groups design was employed in which participants underwent two experimentally-elicited pain procedures, one high- and one low- intensity, with order of exposure counterbalanced. Participants took part in a semi-structured interview about the pain immediately following each pain elicitation.

### Materials

A pneumatically controlled pressure pain apparatus (Dancer Design, UK), with a plastic probe lowered by means of a control dial, was used to elicit pain. The apparatus was fitted with an emergency pressure release button. Three pain questionnaires were also employed: 1) an 11-point numerical rating scale (NRS) for pain intensity (0 =  ‘no pain’, 10 =  ‘worst pain’), 2) [44], [45] an 11-point NRS for pain unpleasantness (0 =  ‘not unpleasant’, 10 =  ‘very unpleasant’) and 3) a Communication Difficulty Questionnaire (CDQ). The CDQ required participants to rate how difficult (from 1 =  Easy to 5 =  Very Difficult) they found it to *verbally* express information about seven aspects of the pain experience (location, intensity, sensation, size, duration, cause, and effects) during the semi-structured interview. The ratings across the seven items are summed to provide a total ‘difficulty’ score ranging between 7 and 35, with a higher score indicating increased difficulty in verbal communication. The CDQ was designed for the purpose of the present study and the seven aspects of pain were empirically derived on the basis of the information contained in speech and co-speech gestures during pain descriptions in a previous study [18]. Internal consistency of the CDQ was assessed using Chronbach's alpha, yielding a value of α = .75, and a bivariate correlation to assess the test-retest reliability revealed a significant correlation between scores across the two testing phases (high intensity and low intensity; *r*(26) = .46, *p* = .019. Although the test-retest reliability is lower than the usual acceptable values (i.e. above ∼.70) it should be noted that this has been calculated on the basis of slightly different administrations of the questionnaire, such that for one administration the questionnaire was completed with reference to difficulty expressing information about high intensity pain, while the other concerned the expression of low intensity pain.

### Procedure

Following instructions about the operation of the pain apparatus, participants aligned the fingernail of the middle finger of their non-dominant hand underneath the probe. Participants then turned the dial to increase the pressure until it reached the specified level on the eleven-point NRS for intensity. For the low intensity condition participants increased the pressure until the pain reached a level that they would rate as a ‘3’ on the NRS, and for the high intensity they increased the pressure until it reached a level they would rate as a ‘7’ on this scale. The intensity levels of ‘3’ and ‘7’ was chosen following piloting to establish the points at which participants judge the feeling of pressure to become painful rather than simply uncomfortable (‘3’) and ‘painful’ without being unbearable (‘7’). Participants kept the pressure at the specified level for thirty seconds before releasing it. The mean level of pressure applied in the low intensity condition was 0.43 bar (*SD* = 0.04; Range  = 0.40–0.50), and 0.53 bar (*SD* = 0.08; Range  = 0.40–0.75) in the high intensity condition (‘bar’ is a metric measurement unit for pressure and one bar is equivalent to 100,000 Pascal, ten newtons per cm^2^, or 2.25 pound-forces per cm^2^). Participants were discouraged from describing the pain experience during the pressure application. Following each pain elicitation, participants took part in a semi-structured interview about the pain. The topic guide for the interviews was based on pain assessment within clinical settings [6], [46], [47] and previous research [18]–[20] and began by asking the participant to describe their pain in as much detail as possible. Follow-up questions were used to elicit additional information about pain location, sensation, intensity, duration, impact and comparisons with other pain experiences. At the end of each interview participants were also asked to complete the three questionnaires (NRS for intensity and unpleasantness, and CDQ). During the interview, the participant and researcher sat opposite each other across a low table at a comfortable conversational distance. The entire procedure was video-recorded split-screen using wall-mounted cameras to give frontal views of the participant and researcher.

### Ethical Considerations

Ethical approval was obtained from The University of Manchester School of Psychological Sciences Research Ethics Committee (Ethics code: 154/07P). All participants provided written informed consent to take part and for the session to be video-recorded. Participants were made aware at the beginning of the study that course credit was not linked to completing the experiment and that they could withdraw at any time and would still receive the study credits.

### Analysis of video data

The analysis focused on participants' responses to the first part of the interview (i.e. where they were asked to describe the pain in as much detail as possible).

*Speech transcription*. The selected portions of the interviews were transcribed verbatim and the total number of words calculated. All speech was included in the word count, including filled pauses (‘er’, ‘um’) and aborted speech. Speech transcription was completed by SR.

*Co-speech gesture identification*. All co-speech gestures produced during the selected portions of the interviews were identified. A co-speech gesture was defined as any movement of the hands, arms, or other body part that occurs alongside speech and is related to that speech [27]. Unrelated movements such as self- or object-adaptors (e.g. playing with the hair or a pen) [48] were not classed as co-speech gestures. Co-speech gesture identification for the entire dataset was carried out by AJW, and to ensure reliability a second coder blind to the experimental hypotheses (SH) independently identified all co-speech gestures in a subset of seven randomly selected interviews from each condition (27% of the data set). Percentage agreement between coders as to which movements constituted co-speech gestures (agreements on individual gestures, divided by total agreements plus total disagreements) was 85% for the high intensity condition and 80% for the low intensity condition, indicating a high level of agreement. From these co-speech gestures, we then identified all those belonging to the subset classed as *representational* co-speech gestures. Representational co-speech gestures [23] contain *semantic information*, such as pointing to the painful area, or rapidly clenching and unclenching the fingers to indicate a throbbing sensation, and were the main focus of the present study. Identification of representational co-speech gestures for the whole dataset was completed by one coder (AJW), while the same second coder (SH) identified representational co-speech gestures in the seven randomly selected files from each condition, yielding Cohen's Kappa values of *k* = .78 (93% agreement; high intensity) and *k* = .63 (85% agreement; low intensity), indicating substantial agreement between the coders [49]. Upon completion of the gesture coding the total number of co-speech gestures overall and the total number of representational co-speech gestures was calculated.

### Statistical analysis

Paired t-tests were performed to compare numbers of words and co-speech gestures and questionnaire scores across the high and low intensity conditions and an alpha criterion level of <.05 (two-tailed) was employed. Paired t-tests were conducted using SPSS Version 20 [50] and post-hoc power analysis was performed using G-Power Version 3.1.3 [51]. Independent sample t-tests comparing the scores of males and females for the number of words and gestures, and pain intensity, unpleasantness and communication difficulty revealed no significant differences (all p>.05) and so the data for males and females was analysed together.

## Results

When talking about high intensity pain, participants used significantly more words, as well as more co-speech gestures (both overall and representational co-speech gestures specifically) than when talking about low intensity pain. Participants also reported significantly more difficulty in verbally communicating about pain (CDQ score) in the high intensity pain condition. Finally, questionnaire measures showed that participants perceived pain to be significantly more intense and unpleasant in the high intensity condition (see Table 1).

**Table 1:** Means (and standard deviations) for questionnaire measures and number of words and co-speech gestures across the two pain conditions (low and high pain intensity).

	Low intensity	High intensity	Paired t-test	Effect size (d)	Observed power
Words	167.46 (84.21)	213.58 (80.45)	*t*(25) = 3.57, *p* = .001	0.56	0.22
All gestures	15.96 (9.17)	23.04 (12.39)	*t*(25) = 2.98, *p* = .006	0.66	0.64
Rep gestures	9.85 (5.88)	15.88 (8.54)	*t*(25) = 3.66, *p* = .001	0.84	0.70
CDQ	12.46 (3.98)	14.38 (4.50)	*t*(25) = 2.21, *p* = .037	0.45	0.54
Intensity	2.94 (0.82)	7.40 (1.25)	*t*(25) = 14.51, *p*<.001	4.31	1.00
Unpleasantness	2.60 (1.55)	6.62 (1.93)	*t*(25) = 11.51, *p*<.001	2.31	1.00

## Discussion

The present study aimed to investigate whether people produce more speech and co-speech gestures when communicating about high intensity compared with low intensity pain. Questionnaire ratings confirmed that people found the high-intensity pain significantly more intense and unpleasant than the low-intensity pain. In line with our hypotheses, when describing high intensity pain, communication was indeed enhanced in comparison to descriptions of low intensity pain. More specifically, people produced both significantly more speech and significantly more co-speech gestures than when describing low intensity pain. These findings show that within an experimental setting, the intensity of acute pain influences the amount of verbal and gestural communication produced. This extends on previous research by indicating that increased pain intensity leads to increases in *spontaneous* verbal communication [41]. In addition, it reveals that, within the domain of visible bodily communication, it is not only the use of nonverbal pain behaviours that increases when pain is more intense [15], but also the use of co-speech gestures. In contrast to pain behaviours, these gestures are rich in semantic content and add important information about a sufferer's pain experience [18]–[20], suggesting that when pain is more intense, people draw on multimodal communication resources to provide richer information about their pain.

The results revealed that participants also rated the high intensity pain to be more difficult to communicate verbally. Despite this, the production of both speech and co-speech gestures increased when pain was more intense. This may suggest that people are motivated to communicate effectively when pain is intense, drawing on speech and co-speech gestures together in an active attempt to overcome the difficulties of speech and communicate their pain as effectively as possible.

The present findings suggest that alongside the semantic content of patients' verbal and gestural depictions of pain, the production of nonverbal pain behaviours such as facial expression, and the results of any pain assessment tools (such as the NRS or multidimensional pain questionnaires), the frequency of speech and co-speech gestures may permit an additional cue to pain intensity. However, in order to assess the utility of the present findings for this purpose it is necessary to go back to the pain communication model we introduced at the beginning of this article and consider how speech and co-speech gesture frequency (B) might influence observers' ability to judge pain intensity (C). While significant, the increases in verbal and gestural behaviour in the high intensity condition could be considered relatively small and it is not yet known whether observers would be able to detect such differences. In the domain of facial expression, research suggests that observers are able to distinguish between real and exaggerated or supressed expressions of pain [52], [53], while research on co-speech gestures indicates that recipients are able to glean the additional information contributed by this modality [54], [55]. However, it is not yet known to what extent the *frequency* of speech or co-speech gestures serve as accurate cues to pain intensity. An important next step is to consider whether and how observers use the extra cue of increased speech and gestures alongside other information about pain intensity (e.g. from the content of speech and the production of nonverbal pain behaviours) to inform their understanding of pain intensity. In doing so it is also necessary to consider the factors that may influence observers' inferences (C) about the pain (A) being communicated (B). For example, factors such as suspected diagnosis, perceptions about patient motivation, and empathy may all influence clinicians' judgements about pain intensity and must be taken into account (see [9] for a more complete discussion of the factors that influence observers' inferences about pain).

The present study represents the first attempt to consider the impact of pain intensity on the production of speech and co-speech gestures and there are a number of limitations that must be acknowledged. Firstly, an experimental pain procedure was used in order to minimise communication differences that may have arisen between the high and low intensity conditions had participants been describing naturally occurring pain that differed in type, past experiences communicating about that pain with others, duration of pain and/or previous treatment. The use of an experimental pain procedure also allowed us to test the same participants across both the high and low intensity pain conditions, minimizing between-participant differences that might have further influenced communication about pain, such as level of worry or catastrophizing about pain. This allowed us to ‘isolate’ the impact of pain intensity on communication for the purpose of this first investigation. However, it is recognised that communication about acute experimental pain cannot be taken as an analogue of the clinical situation. In particular, experimental pain differs in important ways from naturally occurring pain in that it is short-lived, controllable, can be stopped at any time and has a clear cause. In contrast, naturally occurring pain may have no known cause, be a symptom of a potentially serious condition, be ongoing, have an impact on functioning and/or be resistant to attempts at relief, and thus be associated with fear, anxiety and other negative emotions as well as differing motivations to communicate. While participants in an experiment might be motivated to be ‘good’ participants and comply with experimental instructions, the motivation of pain patients and their subsequent communication may differ depending on their personal concerns and goals. For example, communication is likely to differ depending on whether the patient desires reassurance, medication and/or other outcomes such as time off work or a referral to a specialist. Thus, while the present study provides a good starting point for investigations of the impact of pain intensity on verbal and gestural communication of pain, more research is needed to consider the effect of pain intensity on communication in clinical contexts. Such studies will need to measure and control statistically (rather than experimentally) for the influence of variables that might impact on the communication of pain in order to consider the influence of pain intensity. In order for the findings to be generalizable future work should also consider the role of pain intensity in communication about both chronic as well as acute pain experiences.

Secondly, we recognise that the current sample was primarily made up of females, and all participants were interviewed by a female researcher. This limits the generalizability of the findings as it is well known that there are gender differences in the perception [56]–[59] and communication [60] of pain, while the gender combination of patient and healthcare professional (i.e. male-male, female-female or male-female) within clinical settings can also influence communication, with more information sharing occurring in female-female pairings [61]–[64]. Although comparisons of the scores obtained by males and females on each of the measures within the present study (i.e. production of speech and gestures, difficulty communicating about pain and pain intensity and unpleasantness) revealed no gender differences, future work should aim for a more representative sample and should use a variety of gender pairings in order to increase the generalizability of the findings.

It is also acknowledged that the present findings are only applicable within populations with unimpaired verbal and gestural communication. Populations with language or motor impairments may use speech and gestures differently (if at all) and the assessment of pain intensity is likely to be based primarily on non-verbal pain behaviours (including facial expression and postural movements). Thus the frequency of speech and gestures as a possible indicator of pain intensity is not relevant within such populations.

Finally, although the present study indicated that people engage in more verbal and gestural communication when pain is more intense, what has not yet been considered is whether pain intensity influences the *content* of this communication. Both speech and co-speech gestures convey semantic information about the pain experience, such as where it is located, the sensation, duration, intensity and cause, and the physical or emotional impact of pain [18]–[20]. The present findings suggest that more information about pain is being conveyed in the high intensity condition; however, in order to fully appreciate the influence of pain intensity on the communication of pain, future research should consider whether the information conveyed via the verbal and gestural modalities changes as a function of pain intensity. This is particularly important in light of the finding that despite being associated with increased speech and co-speech gesture production, high intensity pain was perceived to be more difficult to communicate verbally than low intensity pain.

In conclusion, this research represents a first step in demonstrating the potential impact of pain intensity on the communication of pain by revealing that people produce more speech and co-speech gestures when communicating about high versus low intensity pain, despite finding intense pain more difficult to communicate. In addition to contributing detailed information about the nature of pain, speech and co-speech gesture production may provide an additional indicator of pain intensity, with possible implications for the treatment and support received by pain sufferers. However, more research is needed to assess the impact of pain intensity on communication in a variety of clinical settings to assess the generalizability of these findings.
